# The Chronic Care Model and its implications for Specialized Outpatient Care

**DOI:** 10.1590/0034-7167-2021-0315

**Published:** 2022-11-28

**Authors:** Francielle Renata Danielli Martins Marques, Grazieli Adrieli Rodrigues Pires, José Luís Guedes Dos Santos, Vanessa Denardi Antoniassi Baldissera, Maria Aparecida Salci

**Affiliations:** I Universidade Estadual de Maringá. Maringá, Paraná, Brazil; II Universidade Federal de Santa Catarina. Florianópolis, Santa Catarina, Brazil

**Keywords:** Health Evaluation, Chronic Disease, Secondary Care, Ambulatory Care, Unified Health System, Evaluación en Salud, Enfermedad Crónica, Atención Secundaria de Salud, Atención Ambulatoria, Sistema Único de Salud, Avaliação em Saúde, Doença Crônica, Atenção Secundária à, Saúde, Assistência Ambulatorial, Sistema Único de Saúde

## Abstract

**Objective:**

to assess the Chronic Care Model implementation in Specialized Outpatient Care and its repercussions for health care.

**Methods:**

qualitative evaluative research, conducted by the Chronic Care Model. We used observation techniques, document analysis and interviews with 21 health professionals from specialized care. Analysis was conducted by triangulation, with the aid of MAXQDA software for initial and focused coding. All ethical aspects were respected.

**Results:**

professionals recognized that the model reorganized service care and administrative practices, presenting positive repercussions for the health of people assisted. The absence of continuing education for service professionals compromised the complete model implementation.

**Final considerations:**

the implementation, even if partial, of the model brings contributions to service improvement. The weaknesses that are still present are compatible with professionals’ difficulty in distancing themselves from the biomedical model.

## RESUMO

**Objetivo:**

avaliar a implementação do Modelo de Atenção às Condições Crônicas na Atenção Ambulatorial Especializada e suas repercussões para a atenção à saúde.

**Métodos:**

pesquisa avaliativa qualitativa, conduzida pelo Modelo de Atenção às Condições Crônicas. Foram empregadas as técnicas de observação, análise documental e entrevistas com 21 profissionais de saúde da atenção especializada. A análise foi conduzida pela triangulação, com auxílio do *software* MAXQDA para as codificações iniciais e focalizadas. Todos os aspectos éticos foram respeitados.

**Resultados:**

os profissionais reconheceram que o modelo reorganizou as práticas assistenciais e administrativas do serviço, apresentando repercussões positivas para a saúde das pessoas atendidas. A ausência de educação permanente para os profissionais do serviço comprometeu a completa implementação do modelo.

**Considerações finais:**

a implementação, mesmo que parcial, do modelo traz contribuições para a melhoria do serviço. As fragilidades ainda presentes são compatíveis com a dificuldade dos profissionais em se distanciarem do modelo biomédico.

## RESUMEN

**Objetivo:**

evaluar la implementación del Modelo de Atención a las Condiciones Crónicas en la Atención Ambulatoria Especializada y sus repercusiones en la atención de la salud.

**Métodos:**

investigación cualitativa evaluativa, realizada por el Modelo de Atención a las Condiciones Crónicas. Se utilizaron técnicas de observación, análisis de documentos y entrevistas a 21 profesionales de la salud de atención especializada. El análisis se realizó por triangulación, con la ayuda del software MAXQDA para la codificación inicial y focalizada. Se respetaron todos los aspectos éticos.

**Resultados:**

los profesionales reconocieron que el modelo reorganizó las prácticas asistenciales y administrativas del servicio, presentando repercusiones positivas para la salud de las personas atendidas. La ausencia de educación continua para los profesionales de los servicios comprometió la implementación completa del modelo.

**Consideraciones finales:**

la implementación, aunque sea parcial, del modelo trae contribuciones para la mejora del servicio. Las debilidades que aún están presentes son compatibles con la dificultad de los profesionales para distanciarse del modelo biomédico.

## INTRODUCTION

Throughout the history of Brazilian public health, important health care models were built, linked to the political and economic moment and to the dominant thinking of the time that, for many decades, was restricted to the biological view^(1)^.

The current configuration of health services has the challenge of reorienting the health care model to cope with chronic conditions. The concept of chronic conditions encompasses characteristics related to duration, symptom control and longitudinality of care, incorporating aspects that go beyond the concept of “diseases”, but which require timely responses from health systems. This concept allows to cover, in addition to chronic diseases, persistent infectious diseases, metabolic diseases, conditions related to life cycles, disorders or deficiencies in the long term, as well as socioeconomic, cultural, environmental, social networks and lifestyle conditions, which present themselves as social determinants for the development of chronic conditions^(2-3)^.

Thus, the Unified Health System (SUS - *Sistema Único de Saúde*) must overcome the biologicist domains, fragmented and mercantilist model, strengthening health practices, with articulation of intersectoral actions aimed at addressing chronic conditions^(1,4)^.

Aiming at breaking up the fragmented care, centered on disease and on medical knowledge, as a result of previous models, the Chronic Care Model (CCM) has been gradually introduced in Brazilian primary, secondary and tertiary health services. Based on the characteristics of the CCM, Risk Pyramid Model (RPM) and Social Determination Model, the CCM was structured anchored in the SUS principles^(2-3)^. It has become a reference by the Ministry of Health (MoH), for the conduct of Health Care Networks (RAS) as part of a policy to combat chronic diseases since 2011^(5)^, proposing the use of soft technologies for its management, aiming at stabilization of chronic conditions^(6)^.

The RAS establishment required an adaptation of services to be operationalized in organizational arrangements of health actions with different technological densities, integrated through technical, logistical and management support systems, to ensure comprehensive care^(5)^. Secondary Care, representing medium complexity and specialized outpatient services, is identified as Specialized Outpatient Care (SOC), according to the nomenclature described by the CCM. SOC is considered by common sense as a bottleneck in the SUS mainly due to the difficulty in accessing services, which generates long waiting lines, and failure to communicate between services^(7)^.

For SOC, the CCM proposes coordinated access by Primary Health Care (PHC) with risk stratification; establishment of joint work between specialists (SOC professionals) and general practitioners (PHC professionals) through training activities, case discussions, obtaining a second opinion; attention focused on multidisciplinary care; evidence-based clinical decisions; care plan as a product of multidisciplinary care; absence of Velcro effect (definitive linking of users to specialized units); and establishment of care function, clinical, educational, teleassistance and research supervision^(3,5,8)^.

As a model referenced by MoH^(5)^, the CCM has been implemented in several Brazilian regions, coordinated by the Brazilian National Council of Health Secretaries (CONASS - *Conselho Nacional dos Secretários de Saúde*)^(9)^. Thus, this study is justified considering the need to analyze the CCM implementation from the perspective of SOC.

Assessments focused on CCM implementation are not very expressive in the scientific literature. Evaluative studies already conducted were conducted from the perspective of PHC^(10-11)^. Thus, knowing the results of CCM assessments, in different locations in the country, can guide managers to better decision-making, improve quality of care and judge the success of implemented public policy.

Given these considerations, the following research question emerged: how do SOC health professionals assess the CCM implementation as a model for health care for people with chronic conditions?

## OBJECTIVE

To assess the CCM implementation in SOC and its repercussions for health care.

## METHODS

### Ethical aspects

The research was approved by the Permanent Commission for Project Assessment and the Standing Research Ethics Committee (COPEP - *Comitê Permanente de Ética em Pesquisa com Seres Humanos*), according to Opinion 4.032.609, in accordance with Resolutions 466/2012 and 510/2016 of the Brazilian National Health Council on research with human beings. All participants signed the Informed Consent Form (ICF). To ensure identity preservation of those involved in the research, they were identified by the letter P (Participant) and a number corresponding to the interview inclusion in the software used.

### Study design

This is qualitative evaluative research^(12)^, which used as a conceptual basis the CCM described in the Care of People with Chronic Diseases Guidelines in the RAS and in the Priority Care Lines^(5)^. We used the Consolidated Criteria for Reporting Qualitative Research (COREQ) protocol as a support tool in relation to the development of qualitative studies.

### Methodological procedures

#### Study setting

The study setting was a SOC located in center-northern Paraná, Brazil, a reference for specialized care for 17 municipalities, which gradually implemented the CCM, starting in 2016, through the RAS for Pregnant Women and Children, Health Mental, Hypertension, Diabetes Mellitus and Elderly. The RAS calls worked every day of the week, with teams made up of 23 health professionals who worked in the morning and afternoon periods. The choice of service for the study is justified by the fact that it is a pioneer in the model implementation in that region.

#### Data source

Initially, the researcher contacted the SOC coordinator, scheduling a date and time for her visit to the service to present the study proposal, which was promptly accepted. The researcher received the list of service professionals, established contact with each one, made a personal presentation and explained the importance and reasons for participating in the research. Inclusion criteria were SOC health professionals who had worked in the RAS for at least three months. Exclusion criteria were not considered. Only one professional did not meet the inclusion criteria, as he had been working in the service for less than three months. Thus, of the 23 professionals who worked in the service, 22 were considered eligible for the research, and were invited to participate, but only one refused to participate in the research, for private reasons. Thus, after formal and voluntary acceptance, participants totaled 21 health professionals, five physicians, four nurses, four psychologists, three nutritionists, two social workers, a physiotherapist, a speech therapist and a pedagogue.

#### Data collection and organization

Data were collected from February to July 2020. Three collection techniques were used: observation with moderate participation; analysis of documents used by SOC for monitoring; and supervision of PHC teams and intensive interview. The combination of different points of view, the diversified training of participants, the use of various sources of information and the use of multiple data collection techniques are in line with the methodological framework^(12)^. All stages of data collection were conducted by a single researcher, a nurse, graduate student, with experience in the techniques used, which made it possible to be closer to the research participants.

Observation with moderate participation occurred in individual consultations and in the administrative routines of SOC professionals. Understand the organizational structure of SOC, the functioning of services offered, aspects related to the care of people with chronic conditions, the perception of the people assisted, the working relationship between SOC professionals and with PHC teams, totaling 30 hours of activity. The researcher recorded in a field diary, her perceptions, results of informal conversations and observations of contradictory behaviors or consistent with the conceptual basis adopted, taking into account the assumptions of the technique used, which provides that the data recorded in a field diary qualify the analysis depth^(12)^.

Document analysis occurred concomitantly with observation, from the verification of the instruments used for stratifying users by PHC, communications to PHC on the completion of monitoring of users in SOC, and electronic spreadsheets shared with PHC containing summary records of attendances. These analyzes allowed us to investigate the frequency of contact with PHC, the records of information sent to PHC, the quality of filling in stratifications and risk groups forwarded by PHC and the quality of records made by SOC. The information collected from the analysis of documents was also recorded in a field diary, meeting the assumptions of data triangulation^(12)^ for qualitative assessment.

In order to carry out the interview, a guide of questions was prepared with information about the professional category, gender, age, education and time working in SOC, in addition to issues related to the approximation of participants with the model theoretical components and adaptations that took place in the service to adapt to the CCM. The guide has undergone face and content validation^(13)^, performed by eight members of the Study and Research in Chronic Conditions Group (GEPECRON). To proceed with validation, the following steps were carried out: deepening of the theme; apparent analysis, related to semantics and clarity of questions; and theoretical analysis of each item in terms of content and relevance^(13)^. With the advance of the COVID-19 pandemic in the country, the service had its services modified in compliance with state technical guidelines. Therefore, the interviews were carried out with the help of virtual technology of applications for videoconferencing and calls, individually, with each participant, respecting confidentiality and privacy, lasting approximately 45 minutes, audio-recorded and fully transcribed by the researcher.

#### Data analysis

The combination of techniques adopted allowed analysis triangulation, providing methodological rigor to guarantee valid and reliable results^(12)^. For data analysis referring to the interviews’ content, the analytical techniques of initial and focused coding were used^(14)^. The initial coding^(14)^ implies transcribing all the material collected, making a rigorous study of analyzed sentences, breaking them down into keywords. At this stage, the data were identified and separated, according to similarities and differences of the dimensions discovered line by line, and 203 codes were revealed. Focused coding^(14)^ requires classification, synthesis, integration and organization of data from the most significant or frequent initial codes or categories. At this point in the analysis, a code synthesis was performed, grouping them by similarity, resulting in the formation of three categories. These steps were carried out with the aid of MAXQDA^®^, version 20.0.8. The records made in a field diary referring to the stages of observation and consultation of documents were manually analyzed after interview coding. They were used to support the understanding of facts and clarification of participants’ statements, supporting value judgments attributed and evaluative analysis.

To make sense of the objective of the investigation, the interpretation of this analytical construction was supported by the conceptual basis adopted^(5)^, using its theoretical precepts to correlate the results obtained on the CCM implementation in the service.

## RESULTS

Twenty-one health professionals participated in the study. Of these, 16 were female, aged between 25 and 61 years, and 18 had at least one graduate and 19 had been in SOC for less than five years.

The results presented addressed how the research participants approached the CCM and understood it, to conduct their professional practices. After the involvement and daily application of CCM precepts by health professionals, there was a need to change the organization of SOC for the proper model implementation and verification of positive repercussions for the health of the people assisted in SOC. In data synthesis, the following categories emerged: *Organization of care in Specialized Outpatient Care after the Chronic Care Model implementation*; and *Repercussions of the Chronic Care Model for the health of people assisted in Specialized Outpatient Care*.

### Organization of care in Specialized Outpatient Care after the Chronic Care Model implementation

**Figure 1:**
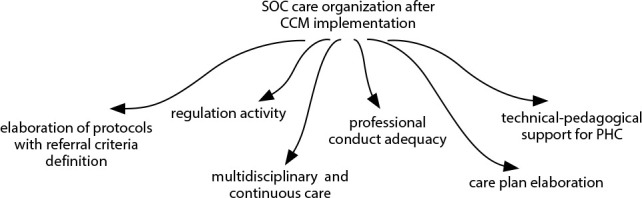
Organization of specialized outpatient care after the Chronic Care Model implementation

This category addresses the reorganization of work required for the CCM implementation in SOC (Figure 1). This change was marked by the approach of professionals to the model’s specificities.

*CCM: Chronic Care Model; PHC: Primary Health Care*

*When I joined, I started to get informed, study a little, read Eugênio Vilaça and I discovered the facets of this care model, which is not simply an outpatient clinic, a consultation, but, rather a longitudinal care where you try to accompany a person throughout their trajectory.* (P06)

*I began to understand on a day-to-day basis, with the demand that was appearing.* (P10)

*I did state training. After a while, the courses started to decrease, but in the beginning, there was a lot.* (P13)

The existence of training was identified in the initial process of CCM insertion in the service, in which the professionals inserted from the moment of implementation participated. It was observed the lack of a continuing education routine for SOC professionals, considered essential due to professional turnover, which can compromise the model implementation for the health care of people with chronic conditions.

*The information we have* [about the CCM] *is information that we go after and get to know* [...] *when a new professional comes here, there is no training. How will they understand this model?* (P20)

SOC care is supported by clinical protocols, prepared according to the guidelines of the state of Paraná and which contain, among other information, the criteria for referring people with chronic conditions to care at SOC.

*These municipalities already have older adults screened by stratification* [...] *and they are sent to us. There is a routing protocol that we created for the service.* (P04)

The insertion of routing criteria caused changes in the form of scheduling for SOC. Prior to the CCM implementation, scheduling was carried out by the PHC itself, without risk stratification and without regulation carried out by a health professional trained to identify priorities. The previous submission to SOC of documentation referring to stratification of users’ risk allowed the insertion of regulation by professionals of specialized care, mainly nurses. The observation recorded in a field diary supported the information obtained through the interviews, making it possible to verify the documents used for risk stratification as well as the flow of these referrals.

*As soon as this protocol* [for referral] *arrives, a nurse makes the regulation. She checks the documentation and the urgency of the case. Those who are more decompensated, with more complications have higher priority.* (P20)

*The service contained a file to store all the stratifications received from PHC, separated by municipality and by Lines of Care*. (field diary notes referring to document analysis)

Despite recognizing that the CCM advocates the assistance of people in SOC based on risk stratification, the assessment pointed out gaps regarding the use of this recommendation. The data obtained from the interviews and observation of consultations revealed that people with simple chronic conditions can have access to SOC, opposing the model’s assumptions, by promoting unnecessary specialized care.

*Not every patient comes with risk stratification* [...] *we find this stratification very complicated and confusing, we do not feel the need for patients to always come with it, because it is difficult to do.* (P10)

*It was observed the reception of people in the service without risk stratification for the Mental Health Care Line, and, even so, they were attended.* (Field diary notes referring to observation)

The insertion of multidisciplinary teams, as directed by the CCM, was positively assessed. These teams, with professionals from different specialties, provide continuous attention to people in the same work shift, minimizing the number of trips to SOC. Each line of care establishes its organization, however it has four to eight professionals involved in the service on the same shift, which favors effective communication between members, since the service does not provide electronic medical records.

*Patients spend with nurse, physician, nutritionist, social worker and physical therapist. We try to optimize the flow, so they don’t get tired.* (P09)

*At least one consultation with each professional they* [users] *need to have.* (P08)

*As we do not have electronic medical records, I created post-it notes for those who have already attended to go ticking. That way, we can organize ourselves better*. (P07)

SOC professionals assessed that the CCM implementation caused changes in their behavior so that they could adapt to the model’s proposal, based on competences and skills developed. The approach to the model particularities caused changes in care for people with chronic conditions, enabling care centered on users and their families, with a focus on educating people for behavioral changes and support for self-care. The field diary records regarding the observations supported the development of a person- and family-centered care.

*The speech therapy and psychology sessions took place together with the families. The professionals were careful to understand the family dynamics, parents’ work routine, income, etc., before suggesting a new care plan.* (Field diary notes referring to observation)

*The verified care plans contained notes on the selection of problems based on the priorities defined in the consultation.* (Field diary notes referring to document analysis)

The development of skills compatible with the CCM assumptions allowed the rupture of a uniprofessional care related to the biomedical model for an integrated care with other health professionals.

*Normally, my consultations with the parents were separate from the children, and here, at the CCM, it is always more with the family together. So, I take care of the family too.* (P21)

*When I take care of children, I can’t just look at her. How will the child be okay if the mother is not? If necessary, I refer this mother to individualized care too.* (P07)

[...] *before, the model was very focused on individual care, you could even have a nutritionist, a nurse, other professionals attending, but it was all individualized, and today it’s all paid in, we talk. I can’t imagine you assisting a person without having a multidisciplinary team.* (P09)

The development of proactive care plans, built in a cooperative manner and with definition of goals, was recognized as a highlight in the reorganization of SOC care.

*We create a complete care plan, with medical, physiotherapeutic, nutritional guidelines, etc., created by multiple hands, both with specialized and basic services.* (P04)

However, the participation of SOC physicians in the moments scheduled for team meetings was considered small. This professional is essential in the discussions of cases, to align the conduct of SOC team and to schedule actions with PHC.

*I think that, in order to improve the issue of physicians’ participation, it would be to put it in the contract, because it has already been tried to talk, establish days to participate in the meetings, and it didn’t work.* (P06)

*Maybe some rules are imposed, like having a team meeting every 15 days.* (P13)

In addition to care issues, the reorganization of SOC also involved technical-pedagogical support for PHC. Educational actions, such as training, meetings and case discussions with PHC, were included in the routine of specialized teams. However, difficulties establishing qualified communication with PHC were assessed as obstacles to carrying out bureaucratic actions, such as supervising PHC activities and monitoring people served at SOC. It is understood that this gap may be related to the attributions recently incorporated in SOC, combined with the difficulties of assimilating the CCM characteristics by all the professionals who make up the points of care.

*A meeting that we have often held in the municipalities is about filling out the referrals. There’s a lot of missing information.* (P08)

*I often call the Health Regional and say that this municipality is difficult to supervise and the Regional goes there, but that doesn’t always solve it.* (P08)

*In order for us to be able to make the patient not just stay here with the Velcro effect, we need the help of primary care, for them to help us monitor this patient, and this is a barrier.* (P10)

The CCM implementation in SOC was marked by assistance and administrative reorganizations, which had a positive impact on improving service quality. However, there are still weaknesses, especially with regard to continuing education professionals and difficulties in taking on supervisory and monitoring functions, as proposed by the CCM.

### Repercussions of the Chronic Care Model for the health of people assisted in Specialized Outpatient Care

**Figure 2:**
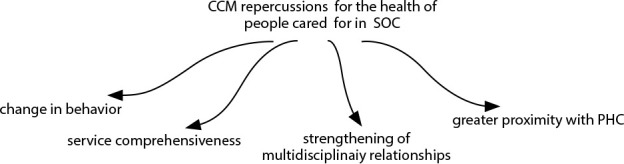
Repercussions of the Chronic Care Model for the health of people assisted in Specialized Outpatient Care

This category presents the CCM contributions to the health of people assisted in SOC, from health professionals’ perspective (Figure 2). For health professionals, the CCM implementation in SOC was assesses as positive for the people who are assisted in this care model. One of the points highlighted were the changes in behavior achieved through multidisciplinary interventions.

*CCM: Chronic Care Model; SOC: Specialized Outpatient Care; PHC: Primary Health Care*

*Countless times we hear from patients: “wow, it made a difference that I received such guidance and reduced the salt in my food. Now my blood pressure is controlling.” These are statements that make us understand that, when it was just the medical consultation, it had no effect.* (P06)

*Patients’ report of gratitude for receiving this attention is very gratifying. Sometimes, they even use it as a comparison, because prenatal care in the municipality is just a physician and nurse, and here they find a nutritionist, psychologist, social worker, speech therapist. Completely changes prenatal! I think we can meet their needs a little more, not only specific prenatal care, but other aspects that occur and that can interfere with pregnancy, such as social vulnerability.* (P12)

The comprehensiveness of services allowed easier access to SOC for people with complex chronic conditions, according to risk stratification, which favors the stabilization of chronic conditions. Document analysis allowed us to understand how referrals were made from PHC to SOC, in order to prioritize the most complex cases.

*In addition to assisting the pregnant woman, after the baby is born, we refer children to high-risk pediatrics. So, if you need a pediatric cardiologist, pediatric endocrinologist, we can already make these referrals.* (P14)

*We already schedule the returns, the exams, we streamline everything for them* [patients] *to have this follow-up faster. This prevents PHC from having to make these appointments, because there it takes.* (P15)

*Before the CCM, patients did not have this ease of access to specialties. Working with the model, he organized the access more and came to add.* (P07)

The strengthening of relations between SOC professionals allowed the broadening of discussions about the planning of health actions.

*For instance, I see a person referred for hypertension, saying that when he gets nervous, the pressure goes up, and I identify a depressed, anxious mood. So, I talk to the physician about my assessment, because this pressure is often not related to a hypertensive disease, but a psychological disease.* (P12)

The service, in accordance with the model theory, made it possible for SOC to be closer to PHC. Discussions of cases, training, use of different tools for communication between teams are described in the CCM literature as prerogatives for adequate model implementation, and in the service assessed, it allowed the development of a link between the two points of care.

*CCM has this quality of letting you know this user in your territory. Therefore, this model makes us strengthen this bond with primary care to be able to take better care of it*. (P10)

The proximity to PHC also revealed weaknesses in the municipalities in responding adequately to the population’s health needs.

*In a given municipality, unfortunately, we do not have all the resources applicable to mental health. So, the flow* [of care] *is often hampered.* (P03)

*I realize that, for the care of adults, the municipalities have more resources, but, for children, it is much more difficult, due to the lack of structure, lack of professionals, even lack of training to know how to deal with the needs of these children.* (P20)

The repercussions in the CCM implementation were assessed as favorable for the stabilization of chronic conditions. However, fragility in resource supply in PHC was recognized as an aspect that needs logistical and financial investment so that it can be more effective in care practice.

## DISCUSSION

The theoretical approach of health professionals with the conceptual bases of the CCM is essential for qualification of SOC, seeking to overcome the previous models whose practices were fragmented and disconnected from the other points of attention of the RAS. Investments in the continuing education of professionals working in SUS are not yet consolidated, failing to encompass essential aspects to work in the scenario of chronic conditions^(15)^.

In a SOC in Ceará, managers and health professionals were trained with the support of the State Health Department, forming a qualified clinical staff to use the CCM, which resulted in advances in the reorganization of SOC^(16)^. In this research, the lack of a routine for the continuing education of employees who work directly with the model is one of the weaknesses identified that does not meet the CCM assumptions. The care of people with chronic diseases implies changes in work processes. Continuing education is essential that goes beyond the traditional models of education for professionals and moves towards educational strategies that value workers, their prior knowledge and their professional experience^(3,5)^. The qualification of professionals through continuing education aims to overcome superficial and fragmented care, which does not prepare the people assisted to self-manage their chronic condition^(6)^.

From the point of view of macroprocesses, changes were observed regarding the reorganization of SOC to implement the CCM. This assessment made it possible to identify the existence of clinical protocols built on the State’s priority guidelines, containing information about access to SOC. Structured clinical protocols and guidelines make up the line of care for chronic diseases, qualifying the demand for SOC and ensuring equity of care in line with the assumptions of regulatory activities^(3,5)^. Other services also identified that the implementation of new work processes for the organization of care between PHC and SOC promotes approximation with new work technologies, such as regulated access through risk stratification^(17-19)^. However, the results of this study pointed to the access of people to SOC without complying with the risk stratification criteria, weakening the adequate CCM implementation and indicating that this process still needs to be consolidated. As already seen in a previous study, people’s access to SOC without linking to risk stratification makes clinical management and stabilization difficult, impairing the CCM organizational structure^(20)^.

The findings of this study revealed a service reorganization with the insertion of a multidisciplinary team to assist people in the same period, thus offering continuous attention. This attention corresponds to the sequential care of different professionals in the same period of care, preventing the person from having to go to the same service on different days, until they are attended by all professionals in the team^(3,8,20)^. Multidisciplinary care is recommended for people with chronic conditions, since the multicausality that surrounds them makes it essential to pay attention with the sum of different knowledge. This result indicated service restructuring to meet the bases of the CCM, since care for people with chronic diseases necessarily involves multidisciplinary care^(3,5)^.

Multidisciplinary care allowed the shared construction of care plans in the service, being in accordance with the CCM. By knowing people’s needs and acting together, SOC and PHC use the same care plan, seeking the same objective: the clinical stabilization of a person with a chronic condition^(3,5)^. The role of shared care plan is to ensure that PHC and SOC professionals are pursuing the same objectives.

The service assessment revealed that the development of teamwork was based on multidiscipline and that interdisciplinary care was incipient. The existence of professionals from various specialties does not necessarily ensure interdisciplinary attention, reiterating the urgency of a continuing education for the service. It is emphasized that the CCM proposes the implementation of multidisciplinary teams that work in an interdisciplinary way^(5)^. Expanding the articulations and integrating the different areas of knowledge around an object of study, in its theoretical and methodological aspects, are certainly one of the greatest challenges today for interdisciplinarity^(21)^. In this study, the results pointed to a multidisciplinary care, but with simple interprofessionality, which allowed the identification of fragmented actions with prescriptive conducts.

The exchange of experiences and information between service professionals was possible due to the reorganization of SOC for moments of discussions and team meetings. The dialogue between health professionals makes it possible to share knowledge, favoring comprehensiveness in several aspects, since no professional alone can ensure a comprehensive approach for people with chronic condition^(22)^. However, the results indicated the little participation of medical professionals in these moments of team meetings, which may indicate a posture rooted in the biological model. There is a need for greater awareness of medical schools to adapt learning scenarios, since higher education institutions are still influenced by the Flexnerian model, distancing medical training from the SUS consolidated practices^(23)^.

The inclusion of technical-pedagogical support for PHC professionals was evaluated as a positive change arising from the CCM. Training provided to PHC professionals, meetings and case discussions were exemplified as part of service reorganization. Other studies found similar changes between the health teams, which were able to establish direct and indirect social relationships that made possible the interaction between the points of the care network^(18-19,24)^.

Weaknesses in the relationship between SOC and PHC, exemplified as the difficulty in reconciling agendas between services, were identified as barriers that make it difficult to carry out supervision and monitoring of people with chronic conditions. These results distance the service assessed from the model theoretical assumptions. For the CCM, SOC professionals need to have time on their agenda for activities that go beyond assistance. Periodic moments for thematic approaches, joint care, discussion of clinical cases in person or at a distance, among others, need to be included in professionals’ agenda^(5)^. Other studies also found difficulties for health professionals to work in accordance with the CCM and continue to develop actions based on the biomedical model, with a more focused performance to meet the demand for medical consultations than for moments of discussion among teams^(18,25)^.

Thus, despite the need to act beyond care activities, disinterest in performing technical-pedagogical support actions, in addition to the activity overload of PHC, is a barrier that compromises actions to monitor users’ health, a reality also found in another study^(25)^. The records in a field diary regarding the documentary analyses allowed the identification of gaps in completing the instruments used for monitoring, indicating weaknesses in the organization of PHC to perform this task, which compromises adequate supervision by SOC.

The CCM implementation repercussions were assessed by participants as favorable for stabilization of chronic conditions. All participants were categorical in stating that, although they still recognize the service limitations, the insertion of multidisciplinarity, continuous care, approach with PHC and the team meetings, even with discreet medical participation, had a positive impact on the health of people assisted at the service. In Iran, research conducted with people with diabetes mellitus identified that the CCM contains the ideal structure for performing user-centered care, with support for the development of self-management in health^(26)^. Other studies have concluded that stabilization of chronic conditions through behavior changes is capable of promoting an increase in people’s satisfaction with their health, with an important institutional gain for care management^(19,27)^. In another SOC in Paraná, after the gradual CCM implementation, significant advances and changes were evidenced in the process of chronic care^(18)^.

Another highlight in the results obtained is the comprehensiveness of services. The understanding that the CCM is favorable for the health of people with chronic conditions is a consequence of providing care of different technological densities in a timely manner. This finding is in line with the comprehensiveness of care points, guaranteeing access to diagnostic and therapeutic resources^(20)^, and is in line with the model, which provides for the guarantee of adequate flows and transitions^(5)^.

The multidisciplinary work, both in care moments and in pedagogical activities, established a closer relationship between SOC members, with the potential to achieve interdisciplinary care. When different knowledge is found, in addition to mutual knowledge anchored in the same work, there is also a personal bond relationship^(3)^. The finding that all knowledge is limited in the face of the complexity of human beings, it mobilizes professionals for joint actions, sharing knowledge and strengthening relationships^(22)^. It is understood that the service has the necessary tools to improve teamwork, qualifying it so that it can achieve interdisciplinary attention, based on a dialogical and horizontal perspective.

Assessment also revealed greater proximity between PHC and SOC teams, made possible by the moments of meetings and case discussions. These findings corroborate another research, which also identified mutual support between teams as a positive factor for achieving comprehensive care for users and their families, even if they are geographically located in different scenarios^(24)^.

The proximity to PHC revealed the fragility in the capacity to offer territorially accessible services in the municipality itself. Decisions related to sus financing often run into misguided political and care decisions that may contradict the current health model^(28)^.

### Study limitations

As limitations, it is noted that this study covered a single specialized care service with a recent insertion of the CCM and that technicians, managers and users were not involved as research participants. Additionally, the short time allocated for observation is recorded, however, it is reinforced that the technique of observation with moderate participation did not correspond to the main method of data collection.

### Contributions to nursing, health, and public policies

This research presents contributions within the scope of health services, academia and nursing, by motivating the proper CCM implementation for the health care of people with chronic conditions. Knowledge about the tools proposed by the model allows for comprehensive and articulated care with the services that make up the care networks, in order to make it possible to break the biomedical model that, for so many decades, has governed care for chronic conditions. The weaknesses identified may support the academy, to strengthen the training of professionals about the model particularities and, consequently, qualify professional practices, especially nurses, due to their ability to provide care and administration.

## FINAL CONSIDERATIONS

The CCM implementation assessment in SOC and its repercussions for the health care of people with chronic conditions was considered by service professionals as satisfactory. As evaluative research, the CCM was partially implemented, with elements that contributed to service modification, but with weaknesses that made its full use impossible.

The reorganization of SOC for the CCM implementation qualified the multidisciplinary care actions, including technical-pedagogical support for PHC professionals. These improvements occurred as a result of the use of soft technologies, with the strengthening of relationships between SOC team members and the joint work with PHC, in accordance with the CCM proposal. Such changes had a positive impact on stabilization of chronic conditions of people assisted at SOC, according to participants.

The aspect that weakened and made it impossible to fully use the CCM was the lack of continuing education for SOC professionals. Programmatic education is capable of strengthening the CCM implementation, as a model that guides professional practices, not only multidisciplinary, but interdisciplinary. The absence of continuing education in the service interfered in the understanding of the model’s specifics by professionals, which contributed to the care of people without stratification criteria and to the small medical participation in the moments of team discussion.

The CCM implementation in its entirety is still a challenge for the service. It is expected that the results presented in this assessment can contribute to a better qualification of care and administrative practices of SOC professionals, in accordance with the theoretical bases of the CCM, bringing improvement in quality of care and the consequent stabilization of the health of people with chronic diseases accompanied by the service.
